# Mitochondrial Energetics and Ca^2+^-Activated ATPase in Obstructive Hypertrophic Cardiomyopathy

**DOI:** 10.3390/jcm9061799

**Published:** 2020-06-09

**Authors:** Maria Lombardi, Davide Lazzeroni, Annalinda Pisano, Francesca Girolami, Ottavio Alfieri, Giovanni La Canna, Giulia d’Amati, Iacopo Olivotto, Ornella E. Rimoldi, Chiara Foglieni, Paolo G. Camici

**Affiliations:** 1 Cardiovascular Research Center, IRCCS San Raffaele Scientific Institute, 20132 Milan, Italy; lombardi.maria@hsr.it (M.L.); lazzeroni.davide@hsr.it (D.L.); camici.paolo@hsr.it (P.G.C.); 2 Department of Radiological, Oncological and Pathological Sciences, Sapienza University of Rome and Policlinico Umberto I, 00161 Rome, Italy; annalinda.pisano@uniroma1.it (A.P.); giulia.damati@uniroma1.it (G.d.); 3 Cardiology Unit, Meyer Children’s Hospital, 50139 Florence, Italy; girolami.fra@gmail.com; 4 Cardiac Surgery Unit, IRCCS San Raffaele Scientific Institute, 20132 Milan, Italy; alfieri.ottavio@hsr.it; 5 Applied Diagnostic Echocardiography Unit, Cardiovascular Department, Humanitas Clinical and Research Hospital, 20089 Rozzano-Milan, Italy; lacannagiovanni.cardio@gmail.com; 6 Cardiomyopathy Unit, Careggi University Hospital, 50139 Florence, Italy; olivottoi@aou-careggi.toscana.it; 7 Institute of Molecular Bioimaging and Physiology, National Research Council (IBFM-CNR), 20090 Segrate-Milan, Italy; rimoldi.ornella@hsr.it; 8 Faculty of Medicine and Surgery, Vita-Salute University, 20132 Milan, Italy

**Keywords:** obstructive hypertrophic cardiomyopathy (HCM), mitochondrial energetics, mitochondrial Complex I (NADH dehydrogenase), mitochondrial Complex V (ATP synthase), reactive oxygen species (ROS) scavenger enzymes, Ca^2+^-activated ATPase

## Abstract

Hypertrophic cardiomyopathy (HCM) is the most common genetic disease of the myocardium associated to mutations in sarcomeric genes, but the link between genotype and phenotype remains poorly understood. Magnetic resonance spectroscopy studies have demonstrated impaired cardiac energetics in patients with HCM, and altered mitochondria were described in biopsies, but little is known about possible perturbations of mitochondrial function and adenosine triphosphate (ATP) production/consumption. The aim of this study was to investigate possible abnormalities in mitochondrial enzymes generating/scavenging reactive oxygen species, and changes in the Ca^2+^-activated ATPases in myocardial tissue from patients with obstructive HCM undergoing surgical myectomy compared to unused donor hearts (CTRL). Methods and Results: Both the amount and activity of mitochondrial Complex I (nicotinamide adenine dinucleotide -reduced form, NADH, dehydrogenase) were upregulated in HCM vs. CTRL, whilst the activity of Complex V (ATP synthase) was not reduced and ATP levels were significantly higher in HCM vs. CTRL. Antioxidant Mn-activated superoxide dismutase (SOD2) and (m)-aconitase activities were increased in HCM vs. CTRL. The Cu/Zn-activated superoxide dismutase (SOD1) amount and mtDNA copy number were unaltered in HCM. Total Ca^2+^-activated ATPase activity and absolute amount were not different HCM vs. CTRL, but the ratio between ATPase sarcoplasmic/endoplasmic reticulum Ca^2+^ transporting type 2 (ATP2A2) and type 1 (ATP2A1), ATP2A2/ATP2A1, was increased in HCM in favor of the slow isoform (ATP2A2). Conclusion: HCM is characterized by mitochondrial Complex I hyperactivity and preserved Ca^2+^-activated ATPase activity with a partial switch towards slow ATP2A2. This data may give insight into the abnormal cellular energetics observed in HCM cardiomyopathy but other studies would need to be performed to confirm the observations described here.

## 1. Introduction

Hypertrophic cardiomyopathy (HCM) is the most common cardiac genetic disease, characterized by an extremely variable clinical phenotype. More than 1400 mutations have been identified, mostly in genes encoding for sarcomeric proteins [1]. Mutation can be demonstrated in approximately 60% of HCM patients and are considered causative [2,3]. A common cellular phenotype induced by HCM sarcomere mutations is an inefficient sarcomere contraction, which is attributed to diverse changes in sarcomere properties including increased myofilament Ca^2+^ sensitivity and triphosphatase (ATPase) activity suggesting increased adenosine triphosphate (ATP) utilization [4]. This is consistent with in vivo studies using ^31^P-magnetic resonance (MR) spectroscopy that have demonstrated impaired cardiac energetics under resting conditions and during exercise in patients with HCM [5,6,7,8]. 

The ATPases are regulators of both mitochondrial and sarcoendoplasmic reticulum Ca^2+^ pumps. Mitochondria directly communicate with sarcoendoplasmic reticulum via a redox signaling interface [9]. Many of the sarcoendoplasmic reticulum mitochondrial Ca^2+^/transporters and signaling proteins are sensitive to redox regulation and are directly exposed to the reactive oxygen species (ROS), oxidative phosphorylation (OXPHOS) by-products that have sensors at the redox signaling interface. Variations in mitochondrial fission and size [10] have been described in biopsies from patients with HCM [11] and oxidative stress-related elevation of 4-hydroxy-2-nonenal (HNE)-modified protein has been shown in the myocardium of HCM patients with left ventricular (LV) systolic dysfunction [12]. Little is known about possible perturbations in mechanisms controlling mitochondrial function and ATP production/consumption [13].

Studies in mutant animal models of HCM support a mitochondrial contribution to some of the heterogeneity of the disease phenotype. Mutant mice at prehypertrophic stage showed preserved mitochondrial function, stimulation of nicotinamide adenine dinucleotide-reduced form (NADH) generation, which fuels respiration and ATP. Conversely a reduction in OXPHOS complex activities was describe in end-stage mice [14,15] Taken together these findings suggested that a hypermetabolic mitochondrial state precedes development of the cardiomyopathy in mice.

Aim of the present study was to ascertain whether there are abnormalities in mitochondrial enzymes associated with changes in the Ca^2+^-activated ATPases in myocardial tissue from patients with obstructive HCM undergoing surgical myectomy compared to unused donor hearts. 

## 2. Methods

Patients with obstructive HCM, diagnosed according to guidelines [16], were prospectively studied and a cohort, selected for surgical myectomy, of therapeutic option in-patients with significant subaortic gradients [17] was enrolled. Inclusion criteria were: unequivocal diagnosis of HCM according to existing guidelines [16] with maximum LV wall thickness ≥ 1.5 cm, age > 18 years, sinus rhythm, presence of severe resting or inducible LV outflow tract obstruction (peak gradient ≥ 50 mmHg), and clinical indication for surgical myectomy, such as the presence of advanced heart failure symptoms (NYHA Class ≥ III), syncope or presyncope due to LV obstruction, exercise hypotension, drug-refractory symptoms, as well as the presence of moderate-to-severe systolic anterior motion-related mitral regurgitation. Exclusion criteria included coronary artery disease, aortic valve stenosis and uncontrolled arterial hypertension (mmHg >140 systolic and/or >90 diastolic) [18]. At surgery, myectomies from the interventricular septum were harvested.

Hearts from unused donors, i.e., discarded from transplantation because of noncardiac technical reasons (e.g., suitable recipient unavailability), were collected at the department of oncological and pathological sciences at Sapienza University (Rome, Italy) and served as controls (CTRL). According to the Italian law, CTRL hearts were provided anonymously, the donors’ age was <60 years, and no data about them were supplied. 

### 2.1. Sample Processing and Histology

Myectomies, harvested immediately during surgery, were cut into 2 mm-thick slices perpendicularly to the endocardium within two hours from collection. Slices were embedded in Killik (BioOptica, Milan, Italy), and frozen in 2-methyl butane/liquid N2 (for confocal analysis and LCM) or snap-frozen in N_2_ (for proteins and mRNA extraction), or processed for paraffin inclusion.

Paraffin blocks were cut and at least two sections were stained with hematoxylin/eosin and Azan–Mallory trichrome stains. Patient biopsies were examined for the presence of the pathologic hallmarks of HCM, i.e., myocyte hypertrophy and disarray, expansion of the interstitial collagen compartment and replacement fibrosis, wall thickening by intimal and medial smooth muscle cell hyperplasia in intramyocardial arteries and narrowing of myocardial capillaries [19].

CTRL biopsies were obtained and processed within two hours from the explant examined under light microscope, to exclude the presence of cardiomyocyte hypertrophy, necrosis or degenerative changes, significant fibrosis, or inflammatory infiltrates.

Serial cryosections (5, 10, or 20 μm thick depending on type of assay) were obtained by a Leica CM1850 cryostat (Leica Microsystems GmbH, Wetzlar, Germany) from frozen slices collected onto slides or in vials for subsequent use. 

Blood samples (10 mL) from patients with HCM and from a group of healthy volunteers (*n* = 11) were withdrawn in EDTA tubes and processed to obtain plasma.

### 2.2. Genetic Testing

Total DNA was extracted from cryosections (kit ID: 69504, Qiagen, Hilden, DE) and 26 genes were analyzed (Supplementary Table S1) by next generation sequencing with MiSeq System (Illumina, San Diego, CA). Variants were classified following guidelines [20] using Cardio-Classifier [21] or InterVar [22].

### 2.3. NADH Dehydrogenase Histochemistry

Frozen sections (5 μm thick) were air-dried, rinsed in phosphate-buffered saline (PBS) 0.05 M pH 7.4 for 5 min at room temperature, then transferred to 1 mM NADH/0.2 mM Nitroblue Tetrazolium/100 mM Tris-HCl buffer pH 8.0 containing 0.2% Triton X-100 solution for a maximum of 1 h at 37 °C to allow the oxidation of NADH and the formation of a stable blue formazan. The reaction was stopped by rinsing the sections in PBS. The sections were analyzed using an Eclipse 55i microscope equipped with a DS-L1 camera (Nikon, Tokyo, Japan) [23,24]. 

The absence of an artifactual signal in the absence of the substrate in the reaction mixture has been verified (Supplementary Figure S1). Optical density of the blue formazan signal was assessed by FiJi software. At least 10 fields from 10× images were analyzed. In these fields the cardiomyocytes areas were selected as regions of interest where the average intensity of signal was measured and normalized versus the area of the region. Interstitium and arteries/arterioles were excluded from the measure.

### 2.4. Quantification of Mitochondrial DNA

Total DNA was extracted from myocardial tissue using the Wizard Genomic DNA purification kit (Promega, Madison, WI, USA). Absolute quantification of mitochondrial DNA was performed by quantitative real-time polymerase chain reaction (PCR) as previously described [25].

### 2.5. Enzyme-Linked Immunosorbent Assay (ELISA)

Tissue amounts of cardiac ATPase sarcoplasmic/endoplasmic reticulum Ca^2+^ transporting type 1 (ATP2A1) and type 2 (ATP2A2) were quantified in protein extracts from HCM and CTRL by specific competitive ELISA (MBS919218, MBS7252760, MyBioSource Inc., San Diego, CA, USA), following the manufacturer’s instructions.

Enzymatic activity of both Cu/Zn-activated superoxide dismutase (SOD1) and Mn-activated superoxide dismutase (SOD2) was determined in freshly prepared tissue homogenate supernatants and pellets, respectively, by a colorimetric activity assay determining the conversion of xanthine oxidase to superoxide in the presence of a substrate, with formation of a colored product to read at 450 nm (EIASODC, Life Technologies Corporation, Carlsbad, CA, USA). Quantification was performed using a standard curve generated with bovine erythrocyte SOD standard.

The activity of mitochondrial (m-)aconitase (conversion of citrate into isocitrate, then to a colored product with a maximum absorbance at 450 nm), the amount of NADH dehydrogenase (sandwich assay, with HeLa cell extract for standard curve) and of ATP synthase (assay designed for cell/tissue lysates, using a capture antibody specific for human ATP synthase, and for measures at 600 nm) were determined by ELISA (ab83459, ab178011, ab124539, all from Abcam, Cambridge, UK). Measurements were performed on an Infinite F200 microplate reader (TECAN Group Ltd., Männedorf, Switzerland). Samples were run in triplicate.

### 2.6. Mitochondrial Complex I and ATP Assays

Mitochondrial Complex I activity was determined by a colorimetric assay (ab109721, Abcam) in mitochondria isolated from frozen myocardial tissues immediately before the test (Mitochondria isolation kit for tissues, ab110168, Abcam). The assay was based on the oxidation of NADH to NAD^+^ and on the simultaneous reduction of a dye, leading to increased absorbance at OD = 450 nm.

ATP content was determined in freshly collected and prepared protein extracts from snap-frozen tissues. (ATP Determination Kit, Molecular Probes, Eugene, OR) as previously described [26]. The assay is based on luciferase requirement for ATP in producing light (emission maximum ~560 nm at pH 7.8).

Samples were run in triplicate and luminescence was measured on a Mitras LB 940 (Berthold, Bad Wildbad, Germany).

### 2.7. ATPase (Fast/Slow) Histochemistry

To visualize fast- and slow-ATPase activities of cardiac cells, two series of unfixed cryosections (5 μm thick) were treated. The assay is based on the different sensitivity of slow/fast ATPase to pH [27,28]. Acid preincubation in 0.1 M sodium acetate buffer at pH 4.6 (10 min at 4 °C) in the presence of 10 mM ETDA inhibited the fast ATPase activity in a series of slides, while the other was incubated with 0.1 M sodium acetate buffer. All the slides were successively incubated in 0.5 mg/mL ATP in 0.1 M sodium acetate buffer at pH 9.4 (11 min at 37 °C), then treated with 2% COCl_2_ for 5 min. Upon rinsing in distilled water, the sections were differentiated in ammonium sulfide for 30s to obtain a brownish-black precipitate of cobalt sulfide. Intensity of the color is associated to activity. The background signal in the absence of ATP in the reaction mixture was verified. (Supplementary Figure S2). Glycerin jelly-mounted sections were analyzed under Eclipse 55i microscope equipped with a DS-L1 camera (Nikon, Tokyo, Japan).

### 2.8. ATPase and Cardiac Muscle Creatine Kinase Activities

Ca^2+^-activated ATPase was evaluated in tissue extracts enriched in myosins [29]. The hydrolysis of inorganic phosphate was measured by spectroscopy [30,31] according to Myosin-ATPase protocol by Sigma-Aldrich. Briefly, tissue protein extracts were obtained and incubated in 200 nM glycine buffer containing 100 mM CaCl_2_ and 50 mM ATP (5 min, room temperature) followed by 10% (NH_4_)_2_MoO_4_ in H_2_SO_4_ solution containing FeSO_4_. Phosphorus solution was used in the standard curve. Absorbance was read after five minutes at 660 nm on an Ultrospec2100pro (GE Healthcare-Amersham Biosciences, Little Chalfont, UK). 

The activity of cardiac muscle creatine kinase (CK-MB) was measured in freshly prepared extracts by a colorimetric assay (68CL-CK-S100, RayBiotech Inc., Peachtree Corners, GA, USA) based on the conversion of creatine to phosphocreatine in the presence of ATP and of a probe. Samples were run in triplicate. The probe’s colored product was read at 450 nm on an Infinite F200 microplate reader and quantified following manufacturer’s instructions.

### 2.9. Statistical Analysis

The Shapiro–Wilk normality test was applied to assess the normality of data distribution. Data from HCM and CTRL were analyzed by *t*-test and correlation between clinical and experimental variables by Spearman test. *p* Values < 0.05 were considered significant. Prism 8.2 was used.

## 3. Results 

### 3.1. Study Population

Seventeen HCM patients were enrolled. Baseline clinical and echocardiographic data are summarized in Table 1 and Table 2, respectively.

Histologic evaluation of myectomies showed features consistent with the clinical diagnosis of HCM in all cases (Supplementary Figure S3). Eight CTRL biopsies were collected and histology showed normal myocardial structure. Due to privacy law, no data about donors were available.

Mutations were demonstrated in 35% of HCM patients (Table 3).

### 3.2. OXPHOS: Expression and Activity of Complex I and Complex V

To investigate potential changes in mitochondrial activity between HCM and CTRL, two enzymatic complexes at the extremes of the OXPHOS respiratory chain were analyzed.

**Figure 1:**
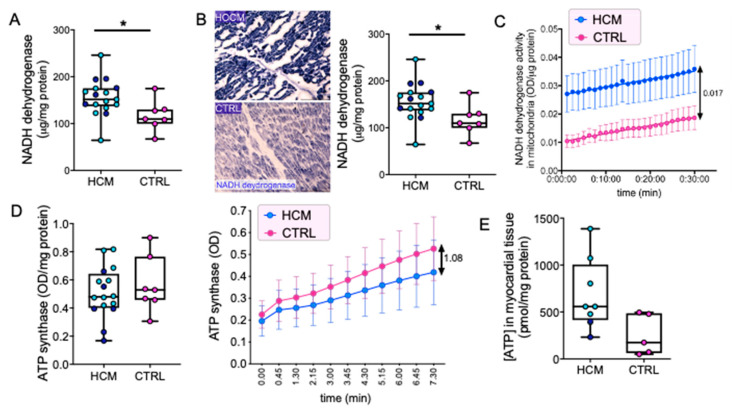
OXPHOS complexes I and V and ATP synthesis. Quantification of NADH dehydrogenase protein amount in myocardial tissue from 17 HCM and seven control (CTRL) samples is shown. (**A**) Representative images of NADH dehydrogenase histochemistry on myocardial cryosections are shown ((**B**), left panels). Blue formazan intensity indicates NADH enzyme activity in cryosections. Semiquantitative evaluation of formazan signal optical density in 10 HCM and six CTRL chosen randomly is presented ((**B**), right graph). Kinetics of Complex I activity (i.e., oxidized NADH) in tissue-isolated mitochondria (**C**) are plotted. Mean optical density values/group ±SEM (17 HCM and 7 CTRL samples) are shown for absorbance read every 1 min. The double arrow indicates the difference between the parallel curves at the end of the measure. Quantitative evaluations of ATP synthase amount ((**D**), left graph) and kinetics ((**D**), right graph) are shown (16 HCM, seven CTRL, three replicates/sample). In ((**D**), right graph), mean optical density values/group ±SEM are shown for absorbance read every 45 s. Double arrow indicates the difference between the fit curves for HCM and CTRL at the end of the measure. Quantification of ATP amount in a subset of myocardial tissues (eight HCM and five CTRL samples) is plotted (**E**). Unpaired *t*-test is applied. Data in (**A**) left, ((**B**,**D**), left) and (**E**) are presented in boxes (min to max) and dots indicate single sample values (mean values from three replicates). Dark blue dots indicate HCM patients with mutation, light blue dots those without. Significant differences are shown as * *p* < 0.05.

A higher amount of NADH dehydrogenase, i.e., Complex I, (*p* = 0.0297) was detected in whole extracts from HCM (Figure 1A). Semiquantitative analysis of NADH dehydrogenase histochemistry showed a stronger signal (*p* = 0.0065) in HCM, but a comparable localization: the signal was intense in cardiomyocytes, weak in vessels, and absent in the interstitium (Figure 1B). Isolated mitochondria demonstrated a significant increase of Complex I activity in HCM (Figure 1C, *p* < 0.0001). 

ATP synthase, i.e., Complex V, level was comparable in whole extracts from HCM and CTRL, and only a trend towards decreased activity was found in HCM (Figure 1D). The amount of myocardial ATP was increased (*p* = 0.0492) in HCM (Figure 1E), but the amount of CK-MB was comparable between HCM and CTRL (Supplementary Figure S4).

### 3.3. Mitochondria: Enzymatic Scavengers and DNA Copy Number

**Figure 2:**
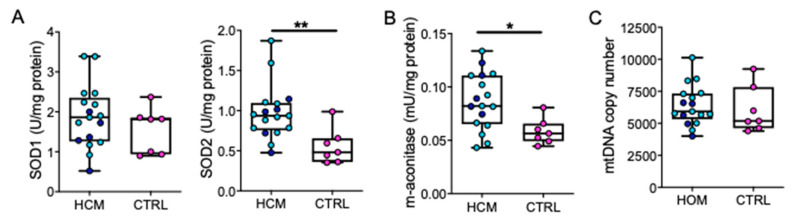
Reactive oxygen species (ROS) scavenging enzymes’ activity and mtDNA quantification of SOD1 (**A**), SOD2 (**B**) and (m-)aconitase (**C**) activities in tissue extracts are plotted, showing high activity of SOD1 in both groups, and the activation of SOD2 and (m)-aconitase in HCM. The absence of difference between HCM and CTRL in total mtDNA copy number into myocardial tissue is displayed (**D**). Data from 17 HCM and seven CTRL samples are presented as boxes (min to max) and dots indicate single sample values (mean values from three replicates). Dark blue dots indicate HCM patients with mutation, light blue dots those without. Unpaired *t*-test is applied. Significant differences are shown as * *p* < 0.05 and ** *p* < 0.01.

The determination of ROS scavengers demonstrated no difference for intracellular Cu/Zn-activated superoxide dismutase (SOD1) activity in HCM vs. CTRL (Figure 2A) while mitochondrial Mn-activated superoxide dismutase (SOD2) (Figure 2B) was significantly higher in HCM (*p* = 0.0069). Furthermore, a significant increase in mitochondrial (m-)aconitase activity was found in HCM (*p* = 0.0143, Figure 2C). No difference in mitochondrial DNA (mtDNA) copy number was found between HCM and CTRL (Figure 2D).

### 3.4. ATPases

**Figure 3:**
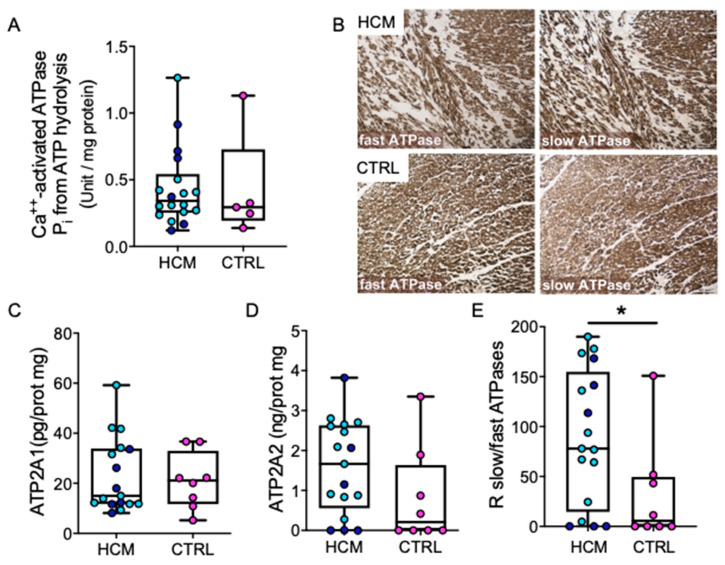
Ca^2+^-activated ATPases. Quantification of P_i_ from ATP hydrolysis by Ca^2+^-activated ATPase in myocardial tissue extracts enriched for myosins is plotted. (**A**) Left atrium from a donor heart and skeletal muscle extracts are used as technical controls. Representative histochemistry images showing the homogeneous distribution of active fast and slow ATPase on cryosections from HCM and CTRL are shown (**B**). Notice the absence of difference in the brown signal intensity, irrespective of the presence of disarray in the HCM field. Tissue contents of fast (ATP2A1, (**C**)) and slow (ATP2A2, (**D**)) ATPase and their ratio measured by ELISA in extracts from HCM (*n* = 17) and CTRL (*n* = 8) are shown (**E**). Data are presented as boxes (min to max) and dots indicate single sample values. Dark blue dots indicate HCM patients with mutation, light blue dots those without. Mann–Whitney test was applied and significant difference is shown as * *p* < 0.05.

The interplay between ROS, ATP, and Ca^2+^-activated ATPase has been described [9]. Ca^2+^-activated ATPase hydrolytic activity was comparable in HCM and CTRL (Figure 3A). ATPase histochemistry showed the fast and slow enzymatic activities in cardiomyocyte bundles and in vessels (Figure 3B), without any difference between HCM and CTRL. The amount of ATP2A2 (SERCA2, slow, Figure 3C) was higher than that of ATP2A1 (SERCA1, fast, Figure 3D) without a significant difference between HCM and CTRL. However, an increased ATP2A2/ATP2A1 ratio was found in HCM (*p* = 0.0404, Figure 3E), indicating a tendency of slow ATPase to prevail.

## 4. Discussion 

The present study demonstrates changes in OXPHOS and ROS scavenger activities in HCM myocardium and a switch from fast to slow Ca^2+^-activated ATPase compared to CTRL. 

Mechanoenergetic uncoupling may play a central pathogenetic role and positron electron tomography (PET) showed that regional work rate of the LV was comparable [32,33]. PET also revealed reduced glucose metabolism but augmented free fatty acid uptake in the hypertrophied septum, and, as free fatty acids give a less energy-efficient fuel, this may contribute to reduced mechanical efficiency [32,33]. Previous papers based on myectomy samples from patients with obstructive HCM [34] have shown that the overarching molecular and energetic features of the disease are comparable and consistent among individuals with different pathogenic mutations in different genes [31]. Phosphorus magnetic spectroscopy showed impaired energy metabolism, demonstrated by reduced PCr/ATP in patients with HCM-harboring mutations in the genes for either β-myosin heavy chain, cardiac troponin T, or MyBPC3, as compared to controls [6]. A mismatch between energy demand and supply has been suggested using ^31^Phosphorus magnetic spectroscopy demonstrated a significant (20%) reduction in resting [PCr]/ATP ratio in HCM patients vs. healthy volunteers, related to the degree of diastolic dysfunction, but not to the degree of hypertrophy, perfusion reserve, or patchy fibrosis [35].

Variability in mitochondrial organization, number, and size was associated with impairment of myocardial contractile and lusitropic reserve in HCM [11]. Impaired cardiac OXPHOS with increased ROS production and enhanced oxidative stress have been described in isolated cardiac muscles and mitochondria from domestic cats with spontaneously occurring HCM [36,37]. Increased Ca^2+^sensitivity of ATPase rate with modification of myofilament proteins was found in Tm-E180G mutant mice [36,37]. Oxidative stress, studied immunohistochemically as expression of 4-hydroxy-2-nonenal (HNE)-modified protein, a major lipid peroxidation product, was elevated in septal biopsy samples from nongenetically profiled patients with HCM compared with control subjects [12]. In line with an increased ROS production, we found that NADH dehydrogenase was increased in both quantity and activity. The latter was counteracted by increased activity of (m-)aconitase, a ROS sensor [38] and mtDNA stabilizer [39], and of antioxidant mitochondrial SOD2 [40]. Conversely, there was no increase in SOD1, another ROS scavenger, suggesting a minor role for this enzyme similarly to human dilated cardiomyopathy [41]. These findings are at odd with those obtained acutely in mice, and human end-stage heart failure (HF). In mice, SOD attenuation was observed in isoproterenol-induced hypertrophy. [42]. Discrepant findings were found in human end-stage HF due to DCM or ischemic cardiomyopathy. In vitro and ex vivo studies showed low (m-)aconitase and SOD2 activities, supporting the oxidative injury as a major determinant of cardiomyocyte dysfunction at post-translational level [43,44]. Conversely, other authors observed upregulated catalase but no difference in SOD isoforms in association to abnormal mitochondrial respiration) [45] or the upregulated SOD2 gene, but downregulated protein activity [46]. As mtDNA is vulnerable to ROS [47], the preservation of mtDNA copy number speaks in favor of ROS quenching by mitochondria in HCM. 

HF can be the consequence of a broad spectrum of different clinical conditions with diverse pathophysiology, which might explain these discrepancies. While classic models of HF are characterized by reduced ejection fraction, HCM is a paradigm of diastolic dysfunction with normal or even super-normal myocardial contractility. Consequently, any a priori assumption that the conclusions of prior studies on HF might be extended to HCM would have been arbitrary [4]. This is why we embarked on the present investigation. The issue of whether the reversal of HF symptoms, observed in >90% of HCM patients undergoing surgical septal myectomy [48] is at least in part related to changes in oxidant/antioxidant enzymes remains to be elucidated.

The novelty of our data on mitochondria resides in the demonstration of the activation of both pro- and anti-ROS mechanisms in HCM, possibly representing an adaptive response, aimed at restraining oxidative stress and energetic loss. These data also underline the intricate balance between ROS generation/scavenging and energy metabolism suggesting a mechanistic difference in the energetic impairment between end-stage HF and HCM. 

Although we cannot exclude ATP recovery in the harvested tissues, the absence of decline in ATP level is in agreement with data in patients with LV hypertrophy secondary to arterial hypertension [49], with previous studies in isolated beating rat hearts in conditions of pressure overload [50], and with the paradigm of inefficient energy usage downstream mitochondria [8]. An altered distance between mitochondria and myofibrils has been reported in several HCM patients [11], representing a possible obstacle for ATP to reach its consumption sites.

Coupling between mitochondria and contractile apparatus or intracellular stores, i.e., sarcoendoplasmic reticulum and myofilaments, involves Ca^2+^-activated ATPases [51,52]. Whether the latter may adapt to mitochondrial changes by activating mechanisms preserving myocardial function remains to be elucidated. Decreased ATP2A2 (SERCA2) was associated to reduced Ca^2+^ uptake by sarcoplasmic reticulum of cardiomyocytes both in experimental models and patients with HF [53]. Increased ATP2A2 activity ameliorated contractility through normalizing the PCr/ATP ratio in animal models of HF (16) and gene transfer to failing rat hearts improved myocardial energetics [54]. The observed switch from fast ATP2A1 to slow ATP2A2 might be finalized at decreasing ATP requirement while maintaining Ca^++^ re-uptake into the endoplasmic reticulum and cell contractility.

### Limitations

We acknowledge several limitations in this proof-of-concept study. We cannot rule out that differences in tissue collection and processing between patients and controls might have affected, at least in part, the results, although similar methods were used previously, supporting the feasibility of enzymatic studies on bioptic samples [55]. Studies of oxidative stress have advanced our understanding of basic mechanisms of disease in the failing heart. Yet, in the two decades since the seminal paper by Dieterich et al, important gaps remain unaddressed, mainly due to the inherent challenges of collecting large series of human myocardial samples and technical difficulties. The ex vivo investigation of human myocardium provides a snapshot of the tissue condition, where most of the enzyme activities are preserved in carefully harvested samples, but highly unstable byproducts such as ROS are difficult to assess. ATP can be measured only within a short time from sample collection and absolute values could be affected by ATP pool replenishment, comparable both in pathological and normal samples (supported by CK-MB activity, similar in both). Any further assessment of the switch from ATP2A1 to ATP2A2 will require a dedicated study by a high-sensitivity proteomic method such as mass spectrometry.

The absence of a relationship between results and patients’ genetic profile may be due, at least in part, to the limited proportion of patients with identified mutation in our HCM cohort. Analyzing individual genotypes separately to identify potential—but likely minor—differences in the molecular and energetic features of the disease would be interesting. However, this would be hardly feasible given the practical implications related to obtaining an adequate number of homogeneous surgical samples. 

The results of the present apply to only to obstructive HCM. Similar studies in nonobstructive HCM are not feasible due to lack of surgical indications.

## 5. Conclusions

This study demonstrates hyperactivity of several myocardial mitochondria enzymes in HCM, suggestive of inefficient energy usage downstream the organelles rather than insufficient supply. The myocardial switch towards slow-twitch Ca^2+^-activated ATPase, more economical in terms of ATP consumption, further supports the defective energy utilization hypothesis of HCM [56].

## Figures and Tables

**Table 1:** Baseline characteristics of hypertrophic cardiomyopathy (HCM) patients.

Demographic Data	*N* = 17
Age (years), M (SD)	59 (9)
Gender (male), *N* (%)	12 (70)
BMI (kg/m^2^), M (SD)	28 (5)
**Clinical data**	
Positive genetic screening, *N* (%)	6 (35)
VUS *N* (%)	3 (17.5)
Family history of HCM, *N* (%)	5 (29)
Family history of SCD, *N* (%)	3 (18)
NYHA ≥ III, *N* (%)	6 (35)
Angina, *N* (%)	1 (6)
Syncope, *N* (%)	2 (12)
Atrial fibrillation (previous history), *N* (%)	3 (18)
NSVT, *N* (%)	1 (6)
**Medical Therapy**	
Beta-blockers, *N* (%)	14 (82)
Antiarrhythmic drugs, *N* (%)	4 (23)
Diuretics, *N* (%)	13 (76)
RAAS-i, *N* (%)	10 (59)

M, mean; SD, standard deviation; *N*, number; %, percentage; BMI, body mass index; VUS, Variants of uncertain significance; SCD, sudden cardiac death; NYHA, New York Heart Association class; NSVT, nonsustained ventricular tachycardia; RAAS-i, renin-angiotensin-aldosterone system inhibitors.

**Table 2:** Echocardiographic data of HCM patients.

Echocardiographic Structural Data	
IVS thickness (mm), M (SD)	20 (5)
PW thickness (mm), M (SD)	14 (3)
IVS/PW ratio, M (SD)	1.5 (0.3)
LV- EDD (mm), M (SD)	45 (7)
LA volume (cc), M (SD)	88 (38)
**Systolic function**	
LV-EF (%), M (SD)	63 (6)
TDI s’ peak (cm/sec), M (SD)	9 (1)
**Diastolic function**	
E peak (cm/sec), M (SD)	56 (25)
A peak (cm/sec), M (SD)	67 (26)
E/A ratio, M (SD)	1.0 (0.8)
DT (ms), M (SD)	153 (55)
IVRT (ms), M (SD)	78 (20)
TDI e’ peak (cm/sec), M (SD)	10 (2)
TDI, a’ peak (cm/sec), M (SD)	12 (4)
E/e’ ratio	7 (4)
**Haemodynamic data**	
PAPs (mmHg), M (SD)	33 (7)
SAM-related LVOT-max gradient at rest (mmHg), M (SD)	76 (30)
Moderate-to-severe mitral regurgitation, *N* (%)	7 (41)

M, mean; SD, standard deviation; *N*, number; %, percentage; IVS, interventricular septum; PW, posterior wall; LV, left ventricular; EDD, end diastolic diameter, LA, left atrial; EF, ejection fraction; TDI, tissue Doppler imaging; s, systolic; E, early filling velocity; A, atrial contraction velocity; DT, deceleration time; IVRT, iso-volumetric relaxation time; PAPs: pulmonary arterial pressure; Max, maximum; LVOT, left ventricular outflow tract.

**Table 3:** Gene variants identified in HCM patients.

ID	Gene	Sequence Variant Nomenclature (HGVS) *	Variant		Publication on Human Gene Mutation Database e **	Sequence Variant Classification (ACMG) ***
			Protein	Type		
1	negative					
2	negative					
3	negative					
4	negative					
5	MYBPC3	c.14581G>A	p.(?)	splice site	Walsh et al. Genet. Med., 2017	Class 5-Pathogenic
	MYBPC3	c.649A>G	p.(Ser217Gly)	missense	Viswanathan et al. PLoS ONE, 2017	Class 3-Unknown pathogenicity
6	MYBPC3	c.772G>A	p.(Glu258Lys)	missense/splice site	Niimura et al. NEJM, 1998	Class 5-Pathogenic
7	MYBPC3	c.3767_3769delCCA	p.(Thr1256del)	inframe Indel	Coppini et al. JACC, 2014	Class 4-Likely pathogenic
8	negative					
9	negative					
10	negative					
11	negative					
12	ACTN2	c.1930 G>A	p.Ala644Thr	missense		Class 3-Unknown pathogenicity
13	negative					
14	MYPN	c.3335C>T	p.(Pro1112Leu)	missense	Bagnall et al. Int. J. Cardiol., 2010	Class 3-Unknown pathogenicity
15	negative					
16	negative					
17	MYH7	c.428G>A	p.(Arg143Gln)	missense	Homburger et al. PNAS, 2016	Class 4-Likely pathogenic
	MYPN	c.3335C>T	p.(Pro1112Leu)	missense	Bagnall et al. Int. J. Cardiol., 2010	Class 3-Unknown pathogenicity

* Sequence variant nomenclature based on Human Genome Variation Society nomenclature (HGVS), ** Human Gene Mutation Database (HGMD^®^) represents an attempt to collate all known (published) gene lesions responsible for human inherited disease, *** sequence variant classification based on American College of Medical Genetics and Genomics (ACMG) guidelines.

## Supplementary Materials

The following are available online at https://www.mdpi.com/2077-0383/9/6/1799/s1, Supplementary Table S1: Analyzed Genes, Supplementary Figure S1: NADH histochemistry: technical contro, Supplementary Figure S2: ATPase histochemistry: technical control, Supplementary Figure S3: Histology, Supplementary Figure S4: Cardiac creatine kinase.
