# Supplementary material for: Mitochondrial Energetics and Ca2+-Activated ATPase in Obstructive Hypertrophic Cardiomyopathy
Source: J Clin Med. 2020 Jun 9;9(6):1799. doi: 10.3390/jcm9061799 (PMC7356244; doi:10.3390/jcm9061799)
Supplement: Supplementary file 1 [file jcm-09-01799-s001.pdf]

Supplementary materials

Mitochondrial Energetics and Ca2+-Activated ATPase in Obstructive Hypertrophic Cardiomyopathy

Supplementary Table 1: Analyzed Genes.

Gene	Transcript	OMIM / Phenotype MIM
ACTC1	NM_005159.4	102540
ACTN2	NM_001103.2	102573
CALR3	NM_145046.3	611414
CAV3	NM_033337.2	601253
CSRP3	NM_003476.2	600824
GLA	NM_000169.2	301500
JPH2	NM_020433.4	613873
LAMP2	NM_002294.2	300257
MYBPC3	NM_000256.3	600958
MYH6	NM_004999.3	160710
MYH7	NM_000257.2	160760
MYL2	NM_000432.3	160781
MYL3	NM_000258.2	160790
MYLK2	NM_033118.3	606566
MYOZ2	NM_016599.3	613838
MYPN	NM_032578.2	608517
NEXN	NM_144573.3	613876
PLN	NM_002667.3	172405
PRKAG2	NM_016203.3	600858
TCAP	NM_003673.3	607487
TNNC1	NM_003280.1	613243
TNNI3	NM_000363.4	191044
TNNT2	NM_001001430	191045
TPM1	NM_000366.5	191010
TTR	NM_000371.3	105210
VCL	NM_014000.2	613255

OMIM: Online Mendelian Inheritance in Man.

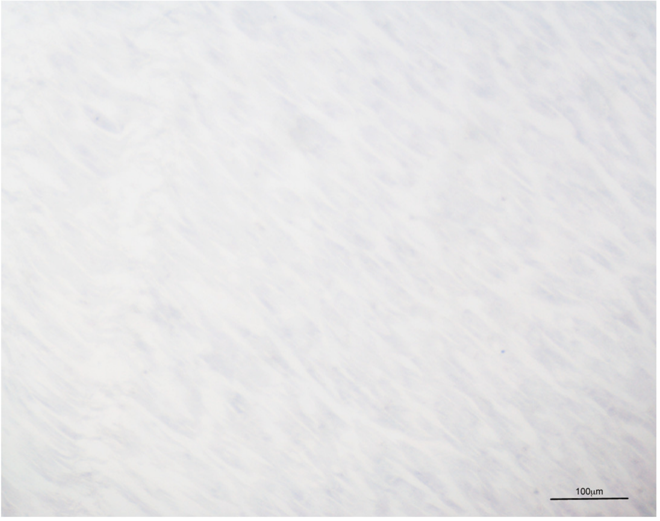

Supplementary Figure 1. NADH histochemistry: technical control. Representative image of myocardial section submitted to NADH histochemistry in the absence of NADH, serving as technical negative control.

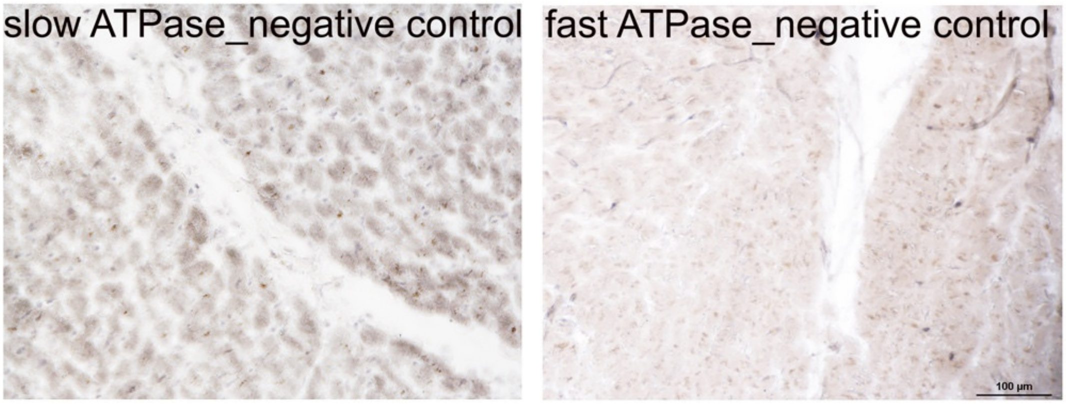

Supplementary Figure 2. ATPase histochemistry: technical control**.** Representative image of myocardial sections submitted to ATPase histochemistry in the absence of ATP in the reaction mixture, serving as technical negative control.

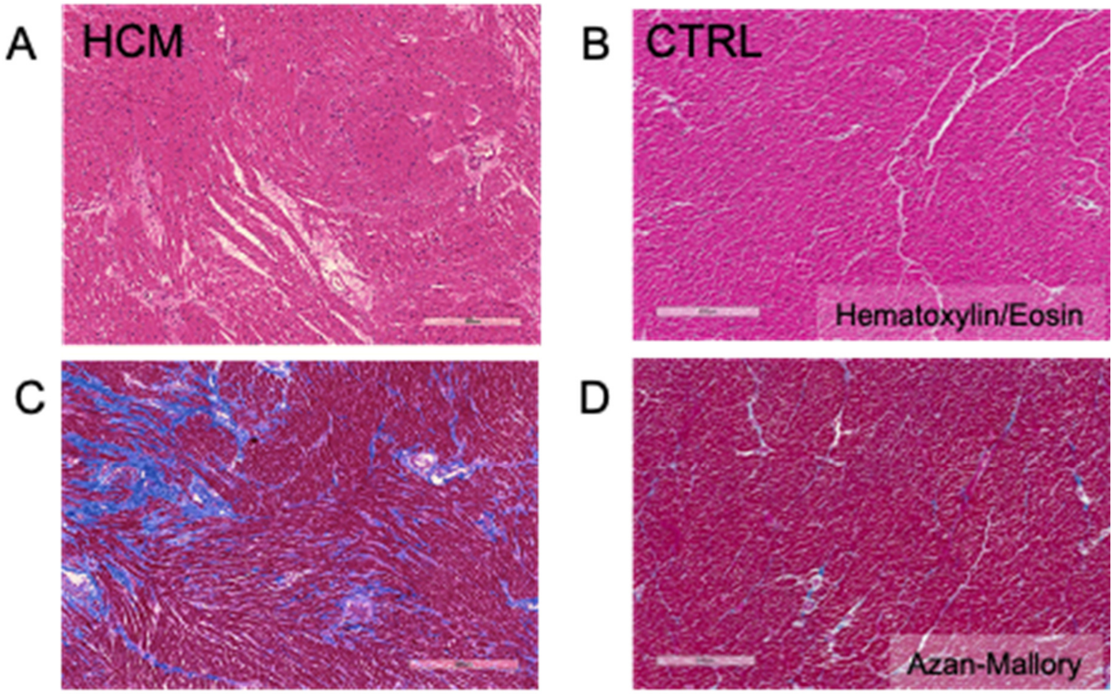

Supplementary Figure 3. Histology. Representative panel of the histologic features of HCM samples, characterized by myofiber disarray (**A,C**) and interstitial fibrosis (**C**). Control hearts (CTRL) show parallel arrangement of myofibers **(B**) and absence of interstitial fibrosis (**D**). Scale bars indicate the magnification.

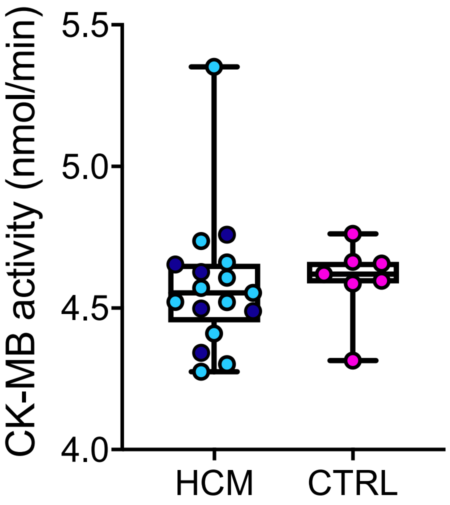

Supplementary Figure 4. Cardiac creatine kinase. Quantitative evaluations of creatine kinase cardiac isoenzyme (CK-MB) amount are shown (17 HCM, 7 CTRL). Data are presented in boxes (5-95 percentile) and dots indicate single sample values (mean values from 3 replicates). Dark blue dots indicate HCM patients with mutation, light blue dots those without.
